# Dynamics of pulmonary hypertension severity in the first 48 h in neonates with prenatally diagnosed congenital diaphragmatic hernia

**DOI:** 10.3389/fped.2023.1164473

**Published:** 2023-06-05

**Authors:** Judith Leyens, Lukas Schroeder, Annegret Geipel, Christoph Berg, Bartolomeo Bo, Lotte Lemloh, Neil Patel, Andreas Mueller, Florian Kipfmueller

**Affiliations:** ^1^ Department of Neonatology and Pediatric Intensive Care, Children’s Hospital, University of Bonn, Bonn, Germany; ^2^ Department of Obstetrics and Prenatal Medicine, University of Bonn, Bonn, Germany; ^3^ Department of Neonatology, Royal Hospital for Children, Glasgow, United Kingdom

**Keywords:** congenital diaphragmatic hernia (CDH), echocardiography, pulmonary hypertension, neonatal ECMO, lung hypoplasia

## Abstract

**Introduction:**

Pulmonary hypertension (PH) is one of the major contributing factors to the high morbidity and mortality in neonates with congenital diaphragmatic hernia (CDH). The severity and duration of postnatal PH are an established risk factor for patient outcome; however, the early postnatal dynamics of PH have not been investigated. This study aims to describe the early course of PH in CDH infants, and its relation to established prognostic markers and outcome measures.

**Methods:**

We performed a monocentric retrospective review of neonates with prenatally diagnosed CDH, who received three standardized echocardiographic examinations at 2–6 h, 24, and 48 h of life. The degree of PH was graded as one of three categories: mild/no, moderate, or severe PH. The characteristics of the three groups and their course of PH over 48 h were compared using univariate and correlational analyses.

**Results:**

Of 165 eligible CDH cases, initial PH classification was mild/no in 28%, moderate in 35%, and severe PH in 37%. The course of PH varied markedly based on the initial staging. No patient with initial no/mild PH developed severe PH, required extracorporeal membrane oxygenation (ECMO)-therapy, or died. Of cases with initial severe PH, 63% had persistent PH at 48 h, 69% required ECMO, and 54% died. Risk factors for any PH included younger gestational age, intrathoracic liver herniation, prenatal fetoscopic endoluminal tracheal occlusion (FETO)-intervention, lower lung to head ratio (LHR), and total fetal lung volume (TFLV). Patients with moderate and severe PH showed similar characteristics, except liver position at 24- (*p* = 0.042) and 48 h (*p* = 0.001), mortality (*p* = 0.001), and ECMO-rate (*p* = 0.035).

**Discussion:**

To our knowledge, this is the first study to systematically assess the dynamics of PH in the first postnatal 48 h at three defined time points. CDH infants with initial moderate and severe PH have a high variation in postnatal PH severity over the first 48 h of life. Patients with mild/no PH have less change in PH severity, and an excellent prognosis. Patients with severe PH at any point have a significantly higher risk for ECMO and mortality. Assessing PH within 2–6 h should be a primary goal in the care for CDH neonates.

## Introduction

Congenital diaphragmatic hernia (CDH) occurs in about 1 in 4,000–5,000 pregnancies (1–4). Although many advances in prenatal and postnatal management have been achieved in recent decades, CDH is still associated with a high morbidity and mortality. Pathophysiologically, three major entities have been identified: lung hypoplasia, pulmonary hypertension (PH), and cardiac dysfunction (5). Postnatal therapeutic options primarily focus on the treatment and correction of PH and cardiac dysfunction. To assess these, point-of-care echocardiography provides an easily and readily accessible tool (5). Approximately one third of CDH infants require extracorporeal membrane oxygenation (ECMO), although its use, outcome, and complication rate vary greatly among institutions (6, 7).

Both, severity and duration of persisting PH are known risk factors for patient outcome in CDH (8). However, all newborns undergo a physiologic decrease in pulmonary vascular resistance (PVR) during early postnatal transition, and using echocardiography a certain degree of right ventricular impairment is commonly seen even in healthy neonates. Nevertheless, the duration of this pulmonary circulatory change is highly variable (9). The correct timing of echocardiographic PH assessment is paramount since a decrease in pathologically elevated PVR occurs in a subgroup of CDH neonates. Recognition of PH and the timing to initiate treatment strategies are important factors in deliberating further therapeutic steps, including ECMO and timing of surgical repair (10).

To our knowledge, no study has been conducted to systematically investigate the dynamics of PH at three defined time points within the first 48 h of life. This study aims to describe the changes in PH within the first 48 postnatal hours, the association of PH with established prognostic parameters, and to evaluate the predictive value of early PH on the outcome parameters need for ECMO and mortality.

## Materials and methods

### Study design

**Figure 1:**
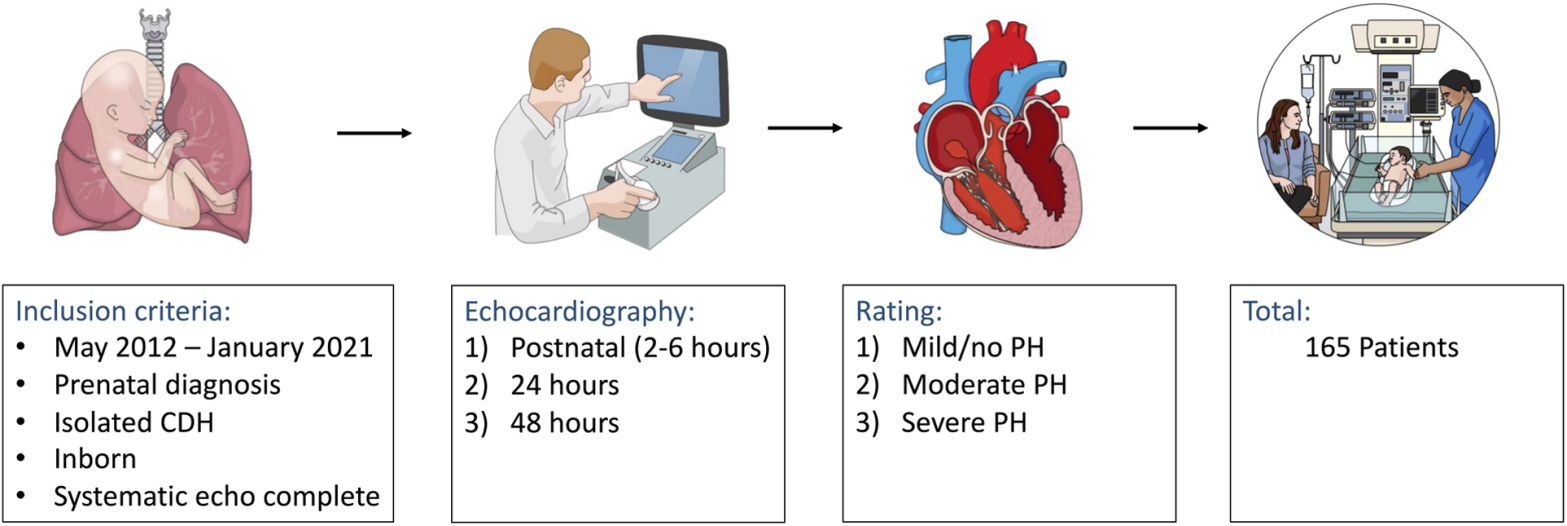
Flow chart of the applied retrospective study design demonstrating data acquisition and analysis of newborn patients with a prenatal diagnosis of isolated congenital diaphragmatic hernia (CDH). PH, pulmonary hypertension.

**Figure 2:**
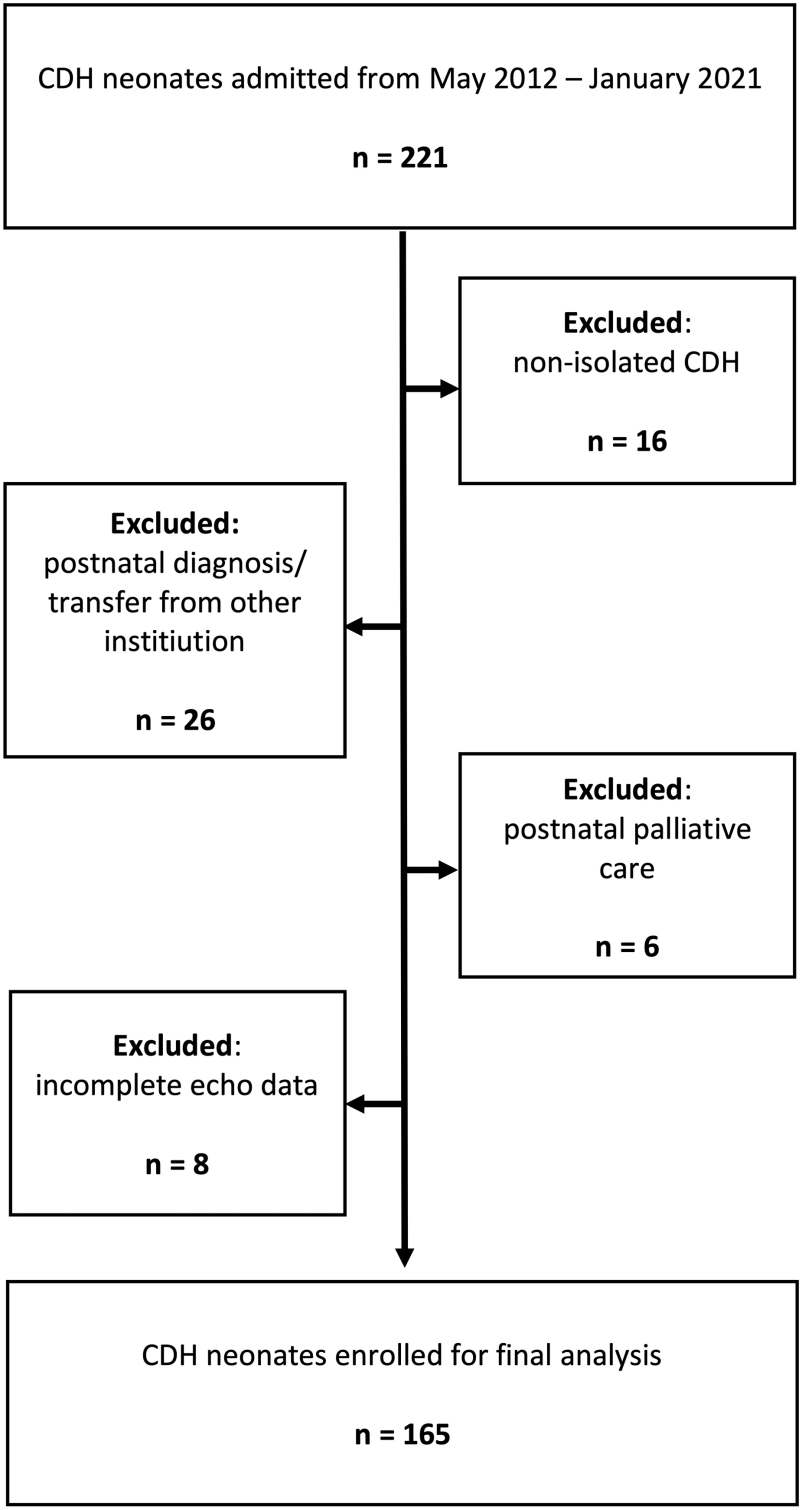
Flow-chart of patient selection demonstrating the number and criteria of excluded patients. CDH, congenital diaphragmatic hernia.

An overview of our study design is shown in Figures 1, 2. Inclusion criteria comprised of: prenatal diagnosis of CDH, isolated CDH, inborn at our institution, and complete echocardiography at the three defined timepoints: 2–6, 24 and 48 h after birth. Patients with non-isolated CDH, postnatal diagnosis of CDH, and outborn patients transferred to our institution after delivery were excluded from this retrospective analysis. Patients with minor associated malformations without any suspected impact on cardiac or pulmonary function (e.g., accessory spleen, cleft lip/palate) were not excluded.

This study has been conducted according to the Declaration of Helsinki guidelines and was approved by the Institutional Ethics Committee (No. 200/22).

### Treatment protocol

Delivery room management was conducted in accordance with the guidelines of the CDH Euro Consortium (11). The patients were intubated after birth and ventilated using lung protective ventilation with an initial fraction of inspired oxygen (FiO_2_) of 1.0. In addition, most patients were treated with inhaled nitric oxide (iNO) with a dose of 5–20 ppm. Cardiac dysfunction and arterial hypotension were treated with dobutamine, milrinone, norepinephrine, and vasopressin, respectively (12). Patients were additionally treated with intravenous sildenafil for PH and levosimendan in case of cardiac dysfunction unresponsive to dobutamine and milrinone (13). Indication for ECMO-therapy was conducted according to the CDH EURO consortium consensus guidelines (11).

### Echocardiography assessment

Echocardiography was performed using an S12-4 sector array transducer on a Philips CX50 CompactExtreme Ultrasound System (Philips Healthcare, Best, The Netherlands). Routinely, echocardiography was performed after delivery room treatment by an experienced neonatologist immediately after admission to the neonatal intensive care unit and initial patient stabilization (2–6 h after birth), followed by repeated exams 24 and 48 h after birth. Echocardiographic assessment of PH included peak tricuspid regurgitation velocity, ductus arteriosus flow pattern, and interventricular shape and position, as proposed by Keller et al. (14). The degree of estimated pulmonary arterial pressure was then classified in relation to the systolic systemic arterial pressure into three groups, ranging from (1) mild/no PH, defined as <2/3 systolic systemic arterial pressure, (2) moderate PH, defined as >2/3 to equal systemic pressure, to (3) severe PH, defined as suprasystemic pressure. The results were read and classified by a single, experienced neonatologist (F.K.) blinded to each patient's identity and outcome.

### Statistical analysis

For statistical analyses, SPSS software (IBM SPSS Statistics for Mac, Version 28.0., IBM Corp, Armonk, NY) was used. Patient groups were analyzed and compared based on their degree and course of PH using Chi-squared, Kruskal-Wallis-test, or ANOVA, as appropriate. Games-Howell-test and Bonferroni correction was used for further post-hoc analysis. Additionally, patients with moderate and severe PH at baseline were divided in two groups, those who showed improvement in PH severity over the observed 48 h (group A) and those, who showed no improvement or worsening PH (group B), and compared. Findings were considered statistically significant in case of *p* < 0.05. For correlation analysis, Spearman- and eta-coefficient tests were used for ordinal/metric or categorical data respectively, as appropriate. Univariate and multivariate analysis using the Cox proportional regression model was performed based on the initial echo data to identify variables independently associated with mortality. Variables that were significantly associated with mortality on univariate analysis were included in the multivariate regression. Results are reported as hazard ratio with 95% confidence interval, standard error and 95% confidence interval.

## Results

### Patient cohort characteristics

In total, 165 newborns fulfilled the above-mentioned criteria and were included in our study. Patient characteristics according to PH severity are presented in Table 1. 15 patients (9%) had minor associated malformations. Sonographic observed/expected lung head ratio (o/e LHR) were available for 162 patients (98%), and magnetic resonance (MR)-tomographic total fetal lung volume (TFLV) data were available for 127 patients (77%). In the majority of patients who required ECMO-therapy the treatment was started within the first 24 h of life (*n* = 48; 81%). The mortality rate for ECMO-patients was 44% (*n* = 26).

**Table 1:** Overview of baseline characteristics for the total patient cohort, and in relation to their pulmonary hypertension (PH)-severity on the initial, 24- and 48-hour exam.

	All infants (*n* = 165)	*P*-value			Initial (*n* = 165)			t24h (*n* = 155)			t48h (*n* = 153)		
		Initial	t24h	t48h	Mild/no PH (*n* = 46; 27.9%)	Moderate PH (*n* = 58; 35.2%)	Severe PH (*n* = 61; 37.0%)	Mild/no PH (*n* = 63; 38.2%)	Moderate PH (n = 55; 33.3%)	Severe PH (*n* = 37; 22.4%)	Mild/no PH (*n* = 85; 51.5%)	Moderate PH (*n* = 51; 30.9%)	Severe PH (*n* = 17; 10.3%)
Sex (*n*=)		.883	.525	.558									
Male	99 (60.0%)				29 (63%)	34 (58.6%)	36 (59.0%)	39 (61.9%)	34 (61.8%)	19 (51.4%)	52 (61.2%)	30 (58.8%)	8 (47.1%)
Mortality (*n*=)		.000*	.000*	.000*									
Survivor	125 (75.8%)				46 (100%)	52 (89.7%)	28 (45.9%)	63 (100.0%)	50 (90.9%)	12 (32.4%)	83 (97.6%)	41 (80.4%)	1 (5.9%)
Non-survivor	40 (24.2%)				0 (0%)	6 (10.3%)	33 (54.1%)	0 (0%)	5 (9.1%)	25 (67.6%)	2 (2.4%)	10 (19.6%)	16 (94.1%)
Gestational age (weeks ± days)	36 + 6 ± 18	.003*	.02*	.018*	38 + 0 ± 11	36 + 5 ± 19	36 + 1 ± 20	37 + 5 ± 13	36 + 5 ± 20	36 + 3 ± 19	37 + 3 ± 15	37 + 0 ± 18	35 + 5 ± 20
Preterm	60 (36.0%)				6 (13%)	24 (41.4%)	30 (49.2%)	14 (22.2%)	21 (38.2%)	18 (48.6%)	21 (24.7%)	19 (37.3%)	11 (64.7%)
Term	105 (64.0%)				40 (87%)	34 (58.6%)	31 (50.8%)	49 (77.8%)	34 (61.8%)	19 (51.4%)	64 (75.3%)	32 (62.7%)	6 (35.3%)
Birth weight (kg)	2.8 ± 0.6	.003*	.072	.051	3.1 ± 0.6	2.7 ± 0.6	2.6 ± 0.6	3.0 ± 0.6	2.8 ± 0.6	2.7 ± 0.6	2.9 ± 0.6	2.8 ± 0.5	2.5 ± 0.7
ECMO (*n*=)	59 (35.8%)	.000*	.000*	.000*	0 (0%)	17 (29.3%)	42 (48.9%)	0 (0%)	29 (52.7%)	30 (81.1%)	13 (15.3%)	31 (60.8%)	15 (88.2%)
Localization (*n*=)		.113	.454	.200									
Left	140 (84.8%)				42 (91.0%)	52 (89.7%)	46 (75.4%)	57 (90.5%)	47 (85.5%)	30 (81.1%)	76 (89.4%)	45 (88.2%)	12 (70.6%)
Right	24 (14.5%)				4 (8.0%)	6 (10.3%)	14 (23.0%)	6 (9.5%)	7 (12.7%)	7 (12.7%)	8 (9.4%)	6 (11.8%)	5 (29.4%)
Liver up (*n*=)	91 (55.2%)	.000*	.000*	.000*	9 (19.6%)	36 (62.1%)	46 (75.4%)	19 (30.2%)	33 (60.0%)	30 (81.1%)	33 (38.8%)	31 (60.8%)	17 (100.0%)
FETO (*n*=)	35 (21.2%)	.003*	.006*	.046*	2 (4.3%)	14 (24.1%)	19 (31.1%)	5 (7.9%)	14 (25.5%)	12 (32.4%)	11 (12.9%)	13 (25.5%)	6 (35.3%)
o/e LHR (%)	40.4 ± 12.9	.000*	.000*	.002*	48.3 ± 12.6	38.9 ± 11.5	35.9 ± 11.9	47.3 ± 12.6	48.5 ± 12.2	33.6 ± 8.7	44.2 ± 13.3	37.8 ± 11.7	34.7 ± 9.6
TFLV (%)	42.5 ± 15.6	.000*	.000*	.000*	54.9 ± 16.9	40.4 ± 13.0	37.2 ± 13.1	52.9 ± 16.2	38.9 ± 13.5	34.7 ± 11.1	48.9 ± 16.6	38.6 ± 12.3	31.5 ± 12.8

Results reported as absolutes (*n*) with % or mean with standard deviation.

* signals significance with *p* < .05.

*P*-values are given for comparison of a baseline characteristic between patients presenting with different PH severity at a specific examination. ECMO, extracorporeal membrane oxygenation; FETO, fetoscopic endoluminal tracheal occlusion; o/e LHR, observed/expected lung head ratio; TFLV, total fetal lung volume.

### Distribution and dynamics of PH in CDH-infants

**Figure 3:**
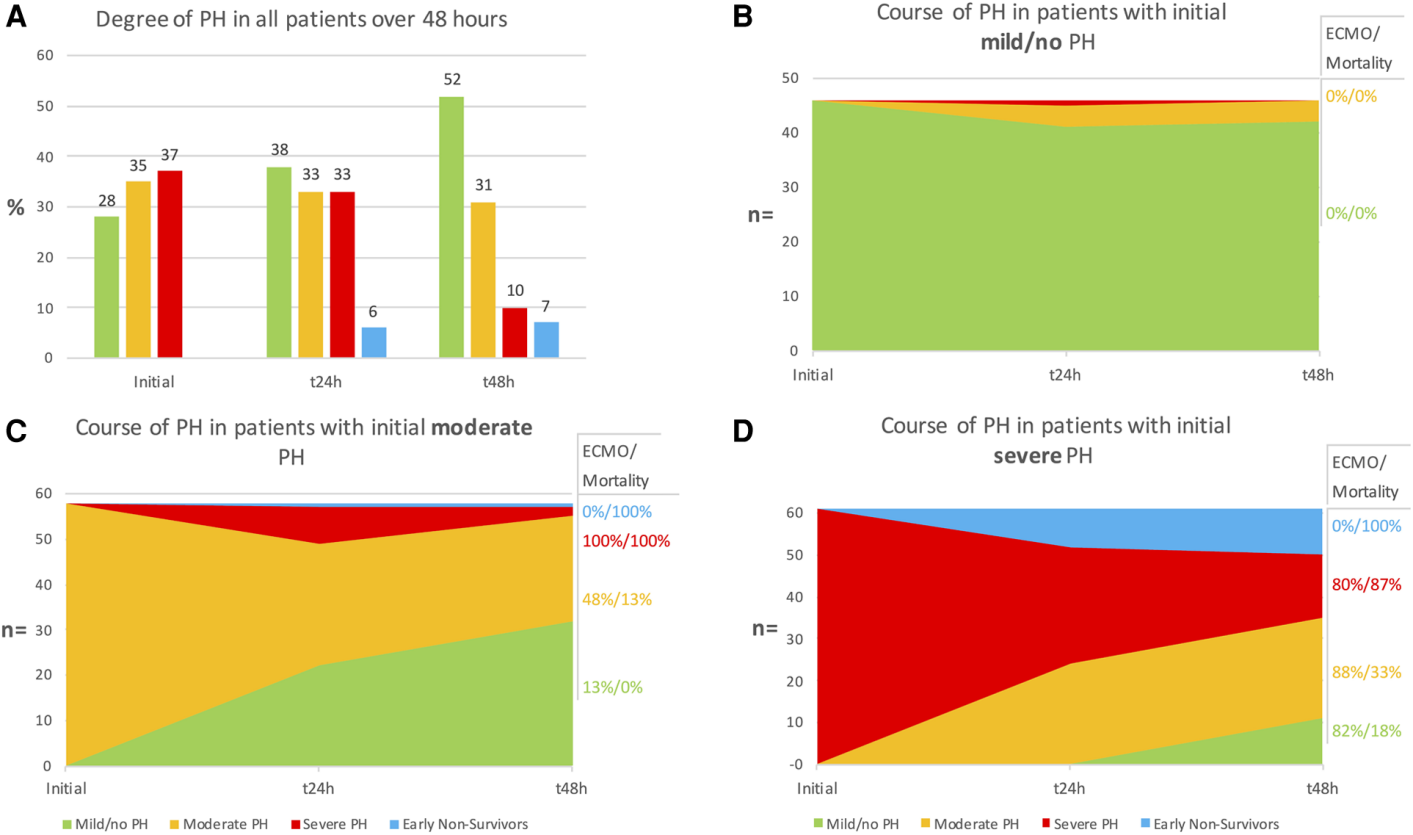
(**A**) Overall distribution of patients showing mild/no, moderate, or severe pulmonary hypertension (PH) at initial, 24- and 48-hour evaluation. (**B–D**) Course of PH in patients with initial mild/no (**B**), moderate (**C**) and severe (**D**) PH. Extracorporal membrane oxygenation (ECMO)-rate and mortality are given in relation to the degree of PH at 48 h.

**Figure 4:**
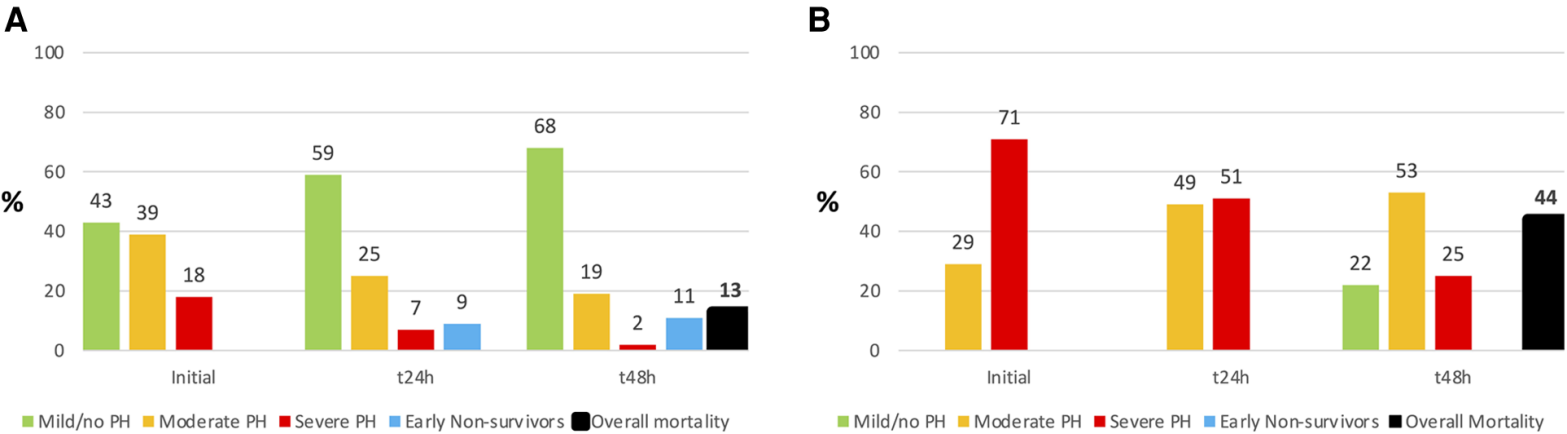
Comparison of pulmonary hypertension (PH) severity in Non-ECMO- (Extracorporal membrane oxygenation; (**A**) and ECMO- (**B**) patients over 48 h. There were no early non-survivors (= death within 48 h) in ECMO patients, but the overall mortality was higher.

The distribution of patients showing mild/no, moderate, or severe PH at the initial, 24-, and 48-hour postnatal evaluation are shown in Figure 3A. The early dynamics of PH over the first 48 h of life depending on the PH severity at baseline are shown in Figures 3B–D. The distribution of PH severity in ECMO and non-ECMO patients is demonstrated in Figure 4. Initially, 46 patients showed mild/no PH (28%). Only 4 of these patients (9%) demonstrated worsening PH. No patient with mild/no PH at baseline required ECMO-therapy, and the survival rate in these patients was 100%. 58 patients (35%) had moderate PH after birth. Of these, 32 patients (55%) showed improvement to mild/no PH, 23 patients (40%) showed no variation and a persistent moderate PH. Two patients (3%) gradually worsened to severe PH. One patient (2%) died 14 h after birth. Overall, 17 patients (29%) with moderate PH at baseline required ECMO-therapy and 6 patients died (mortality 10%). Most infants had severe PH after birth (*n* = 61; 37%). Of these, 35 patients (57%) showed improvement to either mild/no (*n* = 11; 18%) or moderate PH (*n* = 24; 39%) within 48 h. 15 patients (25%) had persistent severe PH. Patients with severe PH at birth had the highest proportion for ECMO-rate (*n* = 42; 69%), early mortality (death within 48 h; *n* = 11; 18%), and overall mortality rate (*n* = 33; 54%). Overall, 48% of all patients demonstrated no variation in PH-severity over the first 48 h. Patients with initial moderate and severe PH demonstrated the greatest variation in their PH-severity (72%).

### Risk factors for PH

Patients with mild/no, moderate, and severe PH were compared according to their characteristics as shown in Table 1 at the initial exam, as well as after 24 and 48 h. There was no difference in sex or side of defect. The results of the post-hoc analysis are shown in Table 2. Overall, patients with mild/no PH differed from the other patient groups in most characteristics. Patients with moderate and severe PH showed striking similarities over the course of 48 h, except for liver position at 24- and 48 h, and the outcome parameters need for ECMO and mortality.

**Table 2:** Post-hoc analysis of characteristics with significance in univariate analysis.

a. Initial	*P*-value			b. t24h	*P*-value			c. t48h	*P*-value		
	Mild/no PH- Moderate PH	Mild/no PH—Severe PH	Moderate PH—Severe PH		Mild/no PH- Moderate PH	Mild/no PH—Severe PH	Moderate PH—Severe PH		Mild/no PH- Moderate PH	Mild/no PH—Severe PH	Moderate PH—Severe PH
GA	.005*	.002*	.981	GA	.116	.077	.985	GA	.578	.055	.197
BW	.004*	.009*	.95	BW	.174	.149	.967	BW	.472	.089	.307
FETO	.013*	.002*	.683	FETO	.033*	.025*	.82	FETO	.193	.200	.749
o/e LHR	.000*	.000*	.657	o/e LHR	.001*	.000*	.092	o/e LHR	.012*	.005*	.539
TFLV	.001*	.000*	.694	TFLV	.000*	.000*	.338	TFLV	.001*	.001*	.211
Liver up	.000*	.000*	.347	Liver up	.003*	.000*	.042*	Liver up	.035*	.000*	.000*
Mortality	.061	.000*	.000*	Mortality	.061	.000*	.000*	Mortality	.013*	.000*	.000*
ECMO	.000*	.000*	.000*	ECMO	.000*	.000*	.001*	ECMO	.000*	.000*	.035*

* signals significance with *p* < .05.

Except for the occurrence of liver herniation, there is no difference in characteristics between patients with moderate and severe pulmonary hypertension (PH), but a striking difference in the outcome parameters extracorporal membrane oxygenation (ECMO)-rate and mortality. GA, gestational age; BW, birth weight; FETO, fetoscopic endoluminal tracheal occlusion; o/e LHR, observed/expected lung head ratio; TFLV, total fetal lung volume.

### Risk factors in early PH severity dynamics

In the comparison of group A and group B, risk factors for progressing or persistent severe PH included right-sided hernia (*p* = 0.016), intrathoracic liver herniation (*p* < 0.001), lower gestational age (GA) (37 + 0 weeks ± 15 days vs. 34 + 6 weeks ± 22 days; p < 0.001), birth weight (BW) (2.8 ± 0.6 kg vs. 2.3 ± 0.7 kg; *p* < 0.001), and TFLV (42 ± 14% vs. 33 ± 10%; *p* = 0.004). The mean o/e LHR was not significantly different between the two groups (39 ± 12% vs. 34 ± 10%; *p* = 0.061).

### Correlation analysis

**Figure 5:**
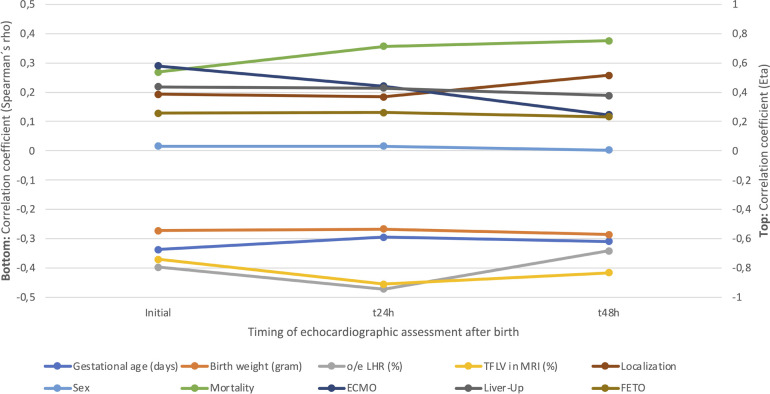
Eta- and Spearmańs correlation for the different characteristics and pulmonary hypertension (PH)-severity at initial, 24- and 48-hour evaluation. o/e LHR, observed/expected lung-head ratio; TFLV, total fetal lung volume; MRI, magnetic resonance imaging; ECMO, extracorporal membrane oxygenation; FETO, fetoscopic endoluminal tracheal occlusion.

The results of the correlation analysis are visualized in Figure 5. There was no significant correlation at any point between the degree of PH and sex, CDH-side, or prenatal fetoscopic endoluminal tracheal occlusion (FETO)-intervention. Only liver position correlated with the degree of PH at 48 h (*p* = 0.037). ECMO-therapy was significantly correlated with the degree of PH at the initial (*p* < 0.001) and second exam (*p* = 0.004), but not at the last exam (*p* = 0.327). Mortality and PH-severity showed a highly significant correlation at all examinations (*p* = 0.001). In addition, PH-severity showed a highly significant inverse correlation with GA (*p* < 0.001), BW (*p* < 0.001), o/e LHR (*p* < 0.001), and TFLV (*p* < 0.001).

### Cox hazard model

Variables associated with mortality on univariate analysis were GA, BW, right-sided CDH, o/e LHR, intrathoracic liver, FETO, biventricular dysfunction, and severity of PH. GA was collinear with BW and therefore not included in the multivariate analysis. Using the multivariate regression model, intrathoracic liver, biventricular dysfunction, and severity of PH, remained independently associated with death (Table 3).

**Table 3:** Univariate and multivariate analysis using the Cox proportional regression model.

	Univariate Analysis				Multivariate Analysis			
Variable	HR	95% CI	SE	*P*-value	HR	95% CI	SE	*P*-value
GA (days)	**0** **.** **980**	**0.968–0.993**	**0** **.** **007**	**0** **.** **003**	0.990	0.973–1.008	0.009	0.281
BW (kg)	**0** **.** **423**	**0.266–0.673**	**0** **.** **236**	**<0** **.** **001**				
Right-sided hernia	**1** **.** **979**	**1.083–3.616**	**0** **.** **308**	**0** **.** **027**	0.886	0.366–2.149	0.457	0.790
o/e LHR(%)	**0** **.** **964**	**0.934–0.996**	**0** **.** **016**	**0** **.** **025**	0.997	0.963–1.033	0.018	0.875
Intrathoracic liver	**8** **.** **161**	**2.502–26.619**	**0** **.** **603**	**0** **.** **001**	**6** **.** **168**	**1.395–27.272**	**0** **.** **758**	**0** **.** **016**
FETO	**2** **.** **237**	**1.177–4.251**	**0** **.** **328**	**0** **.** **014**	1.139	0.577–2.249	0.347	0.707
ECMO	1.938	0.983–3.822	0.346	0.056				
Biventricular dysfunction	**7** **.** **092**	**3.098–16.237**	**0** **.** **423**	**<0** **.** **001**	**2** **.** **609**	**1.059–6.431**	**0** **.** **460**	**0** **.** **037**
Severity of PH	**6** **.** **255**	**2.789–14.030**	**0** **.** **412**	**<0** **.** **001**	**3** **.** **600**	**1.466–8.963**	**0** **.** **465**	**0** **.** **006**

For biventricular dysfunction and severity of PH echocardiography data from the initial examination were used. Variables associated with mortality on univariate and multivariate analysis are highlighted in bold.

GA, gestational age; BW, birth weight; FETO, fetoscopic endoluminal tracheal occlusion; o/e LHR, observed/expected lung head ratio; ECMO, extracorporeal membrane oxygenation; HR, hazard ratio; SE, standard error.

## Discussion

Our study observed equal proportions of mild/no, moderate, or severe PH at the initial postnatal examination in neonates with CDH. This distribution of PH severity after birth in our cohort was similar to previous studies (15). One major finding was the high subsequent variation of PH severity within the first 48 h. Overall, only half of all patients had a consistent degree of PH. None of the patients with mild/no PH at baseline demonstrated a worsening in their PH severity, and these patients had an excellent prognosis with a 100% survival rate and no need for ECMO-therapy. On the other hand, 72% of infants with initial moderate or severe PH had fluctuating PH severity within the first postnatal 48 h. Risk factors for postnatal PH in our cohort included younger GA, lower BW, liver-up, prenatal FETO-treatment, as well as lower prenatal markers (i.e., o/e LHR and TFLV). When evaluating the dynamics of PH-severity, risk factors for worsening or persisting severe PH consisted of younger GA, lower BW, liver-up and lower TFLV. Interestingly, prenatal FETO-treatment and o/e LHR had no impact on the dynamics of PH.

PH is a major contributor to morbidity and mortality in CDH, and an independent risk factor for the need for ECMO-therapy and lasting oxygen-support (15). Besides lung hypoplasia and maldevelopment of pulmonary vessels, the influence of cardiac dysfunction has been increasingly investigated (5, 16). Previous studies demonstrated that prenatal markers such as o/e LHR, intrathoracic liver position, and stomach herniation were correlated with the severity and persistence of PH (8, 17). However, these markers did not predict the occurrence of PH in a recent systematic review (18). In addition, PH has been discussed as an important guiding parameter in the timing of the surgical repair (19).

One issue in assessing the PH severity in neonates arises from the physiologic postnatal circulatory change. In healthy newborns, there is a rapid postnatal fall in PVR, but most infants still have echocardiographic signs of elevated PVR in the first 24–72 h that gradually resolves within 14 days (9, 20). In healthy, non-CDH, preterm infants the PVR seems to fall slower than in term infants (21), and those with hypoplastic lungs are even more at risk of developing persistent PH (22). Based on our data, CDH-infants with a younger GA and lower BW seem to be at a higher risk of severe PH. In our study population, one third (36%) of all patients were born premature, two thirds of these are defined as late-preterms (62.0%). The degree of PH showed a persistent inverse correlation throughout the observed period with GA, BW, and FETO, with a diminishing significance over the observed 48 h. Premature infants had a higher early mortality (*p* < 0.001), resulting in a survival bias. The different dynamics of the decrease in PVR in CDH-infants and those born premature must be considered when distinguishing between physiologically and abnormally elevated PVR and PH. The lack of a universal definition of postnatal PH and timing of assessment are an additional challenge.

Postnatal assessment of disease severity in CDH infants is usually based on a combination of invasive and noninvasive markers, such as preferably preductal arterial blood gas analyses, oxygenation index (OI), laboratory markers like N-terminal-pro-B type natriuretic peptide (NT-proBNP), and echocardiography (23–25).

Previous studies detected PH mostly by using only one single echocardiographic examination within the first 48 h, or post repair, but without exact definition of the age at echocardiographic assessment (8, 15, 26, 27). In our study, CDH-infants were assessed at three defined time points within the first 48 h to describe the dynamics in PH more accurately in the immediate postnatal period. In our opinion, echocardiographic assessment early on the first day of life (e.g., 2–6 h of life) might be most appropriate for risk stratification in CDH neonates. In our cohort, in 81% of all patients managed with ECMO the treatment was already initiated within the first 24 h of life, altering further assessment. Furthermore, postponing echocardiography may lead to an underestimation of PH and missed recognition of cardiac dysfunction. Additionally, CDH infants may experience a “honeymoon”-period with a temporary sufficient pulmonary function before rapid deterioration due to worsening PH.

In our study, when comparing patients with moderate and severe PH, we observed different ECMO and mortality rates despite similar baseline characteristics, except liver position at 24- and 48 h, which we do not believe to be the single reason for the observed difference in these outcome measures (Table 2). In these patients, the difference in PH severity might be aggravated by factors such as acidosis, inflammation, or left ventricular dysfunction. Cardiac dysfunction in particular has been recently shown to be associated with poor outcome in CDH (5, 23, 28, 29). Pathophysiologically, rapid improvement in left ventricular function in the first 48 h might be one of the reasons for improvement of post-capillary PH in some infants (30), and those infants with persisting PH might suffer from predominantly pre-capillary PH. However, data on cardiac function were not available in all patients, and the main focus of our study was to describe the dynamics of postnatal PH.

Limitations of our study include its retrospective nature, as well as the difficulty of interpreting data on the dynamics of PH in patients concurrently receiving pulmonary vasodilators, vasoactive or inotropic agents, or ECMO. The usage of iNO is widely variable among institutions and an ongoing topic of discussion. Studies thus far have failed to show a reduction in mortality rate using iNO, but have shown a short-term benefit in oxygenation among some CDH infants (31, 32). Recent studies even suggest a higher mortality in infants treated with iNO (33), although these patients were also the sickest who were most likely to receive all therapies available, and have a fatal outcome nonetheless. In our institution, iNO is regularly used in CDH patients based on suspected advantages on cellular level as well as ventilation-perfusion-mismatch (34–36). Although its use is controversial, the regular use in our patient cohort contributes to homogeneity and allows for a better patient comparison within our study.

## Conclusion

To our knowledge, this is the first study describing the dynamics of PH in the first postnatal 48 h at three defined time points. None of the patients with initial mild/no PH demonstrated a worsening progression of their PH severity, with an excellent prognosis. There was a high variation of PH severity in patients with moderate and severe PH at baseline. The subsequent dynamics of PH-severity and outcome therefore differed markedly depending on the initial degree of PH. Patients with severe PH at any time had a significantly higher risk for ECMO and mortality. Early echocardiographic assessment at 2–6 h provides information on PH severity in addition to the type of cardiac dysfunction, which might be relevant to guide treatment strategies, such as the initiation of pulmonary vasodilators, inotropes and vasopressors, and might identify high-risk patients with the potential need for ECMO.

## Data Availability

The raw data supporting the conclusions of this article will be made available by the authors, without undue reservation.
